# *In situ* Vaccine Plus Checkpoint Blockade Induces Memory Humoral Response

**DOI:** 10.3389/fimmu.2020.01610

**Published:** 2020-07-24

**Authors:** Claire C. Baniel, Clinton M. Heinze, Anna Hoefges, Elizabeth G. Sumiec, Jaquelyn A. Hank, Peter M. Carlson, Won Jong Jin, Ravi B. Patel, Raghava N. Sriramaneni, Stephen D. Gillies, Amy K. Erbe, Ciara N. Schwarz, Alexander A. Pieper, Alexander L. Rakhmilevich, Paul M. Sondel, Zachary S. Morris

**Affiliations:** ^1^Department of Human Oncology, University of Wisconsin, Madison, WI, United States; ^2^Provenance Biopharmaceuticals, Carlisle, MA, United States; ^3^Department of Pediatrics, University of Wisconsin, Madison, WI, United States

**Keywords:** humoral memory, endogenous antibodies, melanoma, radiation, vaccine, adaptive immunity

## Abstract

In a syngeneic murine melanoma (MEL) model, we recently reported an *in situ* vaccination response to combined radiation (RT) and intra-tumoral (IT) injection of anti-GD2 hu14. 18-IL2 immunocytokine (IC). This combined treatment resulted in 71% complete and durable regression of 5-week tumors, a tumor-specific memory T cell response, and augmented response to systemic anti-CTLA-4 antibody checkpoint blockade. While the ability of radiation to diversify anti-tumor T cell response has been reported, we hypothesize that mice rendered disease-free (DF) by a RT-based ISV might also exhibit a heightened B cell response. C57BL/6 mice were engrafted with 2 × 10^6^ GD2+ B78 MEL and treated at a target tumor size of ~200 mm^3^ with 12 Gy RT, IT-IC on day (D)6-D10, and anti-CTLA-4 on D3, 6, and 9. Serum was collected via facial vein before tumor injection, before treatment, during treatment, after becoming DF, and following rejection of subcutaneous 2 × 10^6^ B78 MEL re-challenge on D90. Flow cytometry demonstrated the presence of tumor-specific IgG in sera from mice rendered DF and rejecting re-challenge with B78 MEL at D90 after starting treatment. Consistent with an adaptive endogenous anti-tumor humoral memory response, these anti-tumor antibodies bound to B78 cells and parental B16 cells (GD2-), but not to the unrelated syngeneic Panc02 or Panc02 GD2+ cell lines. We evaluated the kinetics of this response and observed that tumor-specific IgG was consistently detected by D22 after initiation of treatment, corresponding to a time of rapid tumor regression. The amount of tumor-specific antibody binding to tumor cells (as measured by flow MFI) did not correlate with host animal prognosis. Incubation of B16 MEL cells in DF serum, vs. naïve serum, prior to IV injection, did not delay engraftment of B16 metastases and showed similar overall survival rates. B cell depletion using anti-CD20 or anti-CD19 and anti-B220 did not impact the efficacy of ISV treatment. Thus, treatment with RT + IC + anti-CTLA-4 results in adaptive anti-tumor humoral memory response. This endogenous tumor-specific antibody response does not appear to have therapeutic efficacy but may serve as a biomarker for an anti-tumor T cell response.

## Introduction

Immunological memory is a critical component of adaptive immunity and may be essential for the long-term success of cancer therapies that prevent the growth of metastases and disease recurrence (1, 2). Immunotherapies are a type of cancer therapy intended to boost anti-tumor immune response (3). In order to maximize the therapeutic effectiveness of immunotherapies it may be beneficial to combine multiple agents that guide immune function via complementary mechanisms. With such drug combinations, toxicities may be increased. One approach to minimize this risk has been to employ intratumoral (IT) routes of immunotherapy delivery (4). Such approaches make use of the capacity of a local adaptive immune system to ultimately result in a systemic anti-tumor response. The effectiveness of immunotherapies, including IT delivered agents, may be bolstered through combined modality approaches. Locally administered radiotherapy (RT) is one such treatment that can improve immunotherapy efficacy and induce systemic antitumor responses in patients via an *in situ* vaccine effect (5, 6). *In situ* vaccination is a therapeutic strategy that seeks to transform a patient's own tumor into a nidus for enhanced tumor-specific antigen presentation with the intention of stimulating and diversifying a systemic antitumor adaptive immune response (7, 8). We previously reported that the combination of RT with IT injection of the immunocytokine (IC), hu14.18-IL2, provides a potent antitumor therapy for the GD2 antigen expressing B78 melanoma (9). Hu14.18-IL2 is a synthetic fusion protein consisting of an anti-GD2 tumor-specific antibody genetically fused with IL2, an immune-stimulating cytokine. With this treatment regimen, we observed an *in situ* vaccination effect resulting in complete tumor regression in 71% of mice (9). Mice that experienced complete tumor regression after treatment with our dual RT + IT-IC therapy demonstrated a tumor-specific memory T-cell response. This T-cell response enabled rejection of the parental tumor lines that lacked the GD2 antigen targeted by IC, consistent with the generation of adaptive anti-tumor immunity (9). The combination of this therapy with immune checkpoint inhibition (hu14.18-IL2 + RT + anti-CTLA-4) further amplified anti-tumor responses and resulted in greater tumor regression and improved animal survival when compared to IC, RT or anti-CTLA-4 given alone, or dual combinations of: (1) RT + IC, (2) RT + anti-CTLA-4, or (3) IC + anti-CTLA-4 (9).

Although T cell responses are crucial for provoking a memory anti-tumor response, the importance of humoral responses during treatment and in the memory phase of anti-cancer *in situ* vaccine regimens have not been investigated thoroughly. Given the potent antitumor efficacy of our RT + IT-IC + anti-CTLA-4 *in situ* vaccine regimen, we hypothesized that such a powerful immune provoking therapy might also be priming a humoral anti-tumor response. Memory B cells and the antibodies they produce compose a major part of immune memory (10–12), enabling persistent recognition of an antigen without continuous stimulation (12). However, the role of B cells in the response to many cancer immunotherapies has not been clarified. Some reports indicate that B cells enhance anti-tumor response through roles in antigen presentation and co-stimulation of T cells (13, 14). In contrast, other studies highlight the roles of B regulatory cells, which may antagonize the anti-cancer response (15, 16). In this report, we evaluate the endogenous antitumor antibody response generated by a combined modality *in situ* vaccine regimen, RT + IT-IC + anti-CTLA-4.

## Methods

### Treatment of B78 Tumor Bearing Mice With RT + IT-IC + anti-CTLA-4

Mice were injected with 2 million B78-D14 (B78; a GD2+cell line derived from B16 melanoma, Scripps Research Institute, La Jolla, CA, 2002) cells in the right flank as previously described (9, 17). When tumors reached ~200 mm^3^, mice were shielded with custom designed lead jigs and tumors were radiated to a dose of 12 Gy on what was defined as D1 of treatment. Mice subsequently received 200 μg anti-CTLA-4 IP on D3, 6, and 9 and 50 μg IT-IC on D6-10 of treatment. Tumors were measured twice weekly with digital calipers and survival endpoint was declared if any tumor was >20 mm in any direction. Tumor volume was calculated as length × width^2^/2. Disease-free mice were tested for immunologic memory by a re-challenge in the opposite flank with 2 million B78 cells on treatment D90, or a minimum of 30 days after flank tumor was undetectable. Serum was collected immediately prior to and 10–12 days after re-challenge.

### Detection of Anti-tumor Antibodies Using Flow Cytometry

To assess for the presence of anti-tumor antibodies in treated mice, blood was collected at multiple time points in tumor bearing mice and mice rendered tumor free by treatment. Serum components were isolated and frozen at −80°C until ready for analysis, at which point serum was thawed and co-incubated with the cells being labeled. Labeled cells were washed and tumor bound antibody was detected using secondary antibodies [goat anti-mouse IgG-APC (405308; Biolegend), anti-mouse IgG1-FITC (1070-02; Southern Biotech), anti-mouse IgG2b-FITC (1090-02; Southern Biotech), anti-mouse IgG2c-FITC (NBP2-68518; Novus)] and a live dead vitality stain (DAPI). Target cells included: murine melanoma B16-F10 (B16; ATCC, 2005), Panc02 pancreatic tumor cells (NCI, 2012). Some B16 melanoma cells and Panc02 pancreatic cancer cells were also transduced to express GD2 (B16-GD2+ and Panc02-GD2+) using a retroviral vector that encodes the GD2 mini-operon [MP9956:SFG.GD3synthase-2A-GD2synthase plasmid; a kind gift from Prof. Martin Pule from University College London; (18)]. After transduction, B16-GD2+ and Panc02-GD2+ cells were labeled using PE-conjugated anti-GD2 (14G2a-PE, 357304, BioLegend) and sorted on a BD FACSAria flow cytometer to isolate GD2+ cells with the highest GD2 expression. GD2 expression was confirmed by flow cytometry prior to use. CD16 levels were assessed using anti-mouse CD16-PE (BD Pharmingen 553145). Cell line authentication was performed per ATCC guidelines using morphology, growth curves, and mycoplasma testing (19).

### Cytotoxicity Assays

Antibody dependent cellular cytotoxicity (ADCC) was tested using ^51^chromium-release assays. B16 GD2+ melanoma cells were labeled with ^51^chromium and incubated with murine effector cells. To obtain murine effector cells, 50,000 IU of recombinant human interleukin-2 (rIL-2) (23-6019; TECIN) were injected IP daily in naïve C57BL/6 mice for 3 days prior to the experiment. On the day of the experiment, the mouse's spleen was removed, and splenocytes harvested as effector cells. Five microliter of serum containing endogenous antibodies or 1 μl hu14.18K322a (anti-GD2 mAb, positive control, 1 mg/ml) was added to 5 × 10^4^ target cells in the presence of various effector:target ratios. Antitumor functionality was quantified by percent cytotoxicity and is represented by the mean and standard error of the mean (SEM). Complement dependent cytotoxicity (CDC) was also measured; a similar protocol as ADDC was followed, however rabbit complement (Cappel 55866) was added in place of effector cells. ADCC and CDC were measured using a gamma counter (Packard COBRA) to quantify release of ^51^chromium. Analysis was performed as previously described (20).

### Depletion of B Cells With Anti-CD20 or Anti-CD19 and Anti-B220 Antibody

Murine cell depletion was conducted using anti-CD20 or anti-CD19 and anti-B220 in combination. Anti-CD20 (a generous gift from Provenance Biopharmaceuticals) was administered by intraperitoneal (IP) injection on D0 prior to treatment initiation, and D13 after the completion of therapy. Blood was collected and the absence of B cells was determined by analysis of PBMCs using flow cytometry (Supplemental Figure 1). Anti-CD19 (Bio-Xcell 1D3) and anti-B220 (Bio-Xcell RA3.3A1/6.1) were used to deplete B cells, including established plasma B cells (21); antibodies were given starting 21 days prior to immune memory re-challenge (on D69) with B78 cells in disease-free mice, and then every 5 days, until D10 after rechallenge, in order to ensure depletion of all B cells, as previously reported (21) (Supplemental Figure 2). Given half-lives of mouse IgG are at most 8 days (IgM 2 days, IgG1, IgG2a, and IgG3 6–8 days, IgG2b 4–6 days), we began our B cell depletion 21 days prior to re-challenge with B78 tumor cells (22).

### Pre-treatment of B16 GD2+ Luc+ Cells With Serum

Mouse B16 melanoma cells were transduced to express GD2 and luciferase (B16-GD2-luciferase) using a retroviral vector that encodes GD2 and GD3 synthases together with firefly luciferase (MP9957); a kind gift from Prof. Martin Pule from University College London). B16 GD2+ luc+ cells were incubated for 1 h in either naïve serum alone, disease free serum, or 20 μg/mL ch14.18 in naïve serum at a concentration of 50,000 cells/μL serum. Cells were injected IV via tail vein in C57BL/6 mice for each group. On day 28 after tumor engraftment, mice were imaged by a Perkin Elmer IVIS Spectrum for the presence of B16luc+ lung metastases. Survival was monitored until D60 following injection. Animals were sacrificed when they displayed signs consistent with high metastatic burden or whenever recommended by an independent animal health monitor.

### Statistical Methods

Tumor volume was plotted over time and displayed as mean ± standard error. Significance was determined using a paired *T*-test between relevant groups at a predetermined single final set point. Survival curves were generated using the Kaplan-Meier method and compared using log-rank tests. Re-engraftment rates were calculated using chi-square test. Results of flow cytometry, ADCC assays, and CDC assays were evaluated using ANOVA with post-testing done using two-sample *t*-tests. *P* < 0.05 were considered significant and are indicated in figures as ^****^p < 0.00001, ^***^p < 0.0001, ^**^p < 0.001, ^*^p < 0.05, NS = non-significant (*p* ≥ 0.05). Analyses were performed using JMP and SAS software (SAS Institute, Cary, NC).

## Results

### Tumor-Specific, Adaptive Humoral Response to an RT + IT-IC + anti-CTLA-4

**Figure 1:**
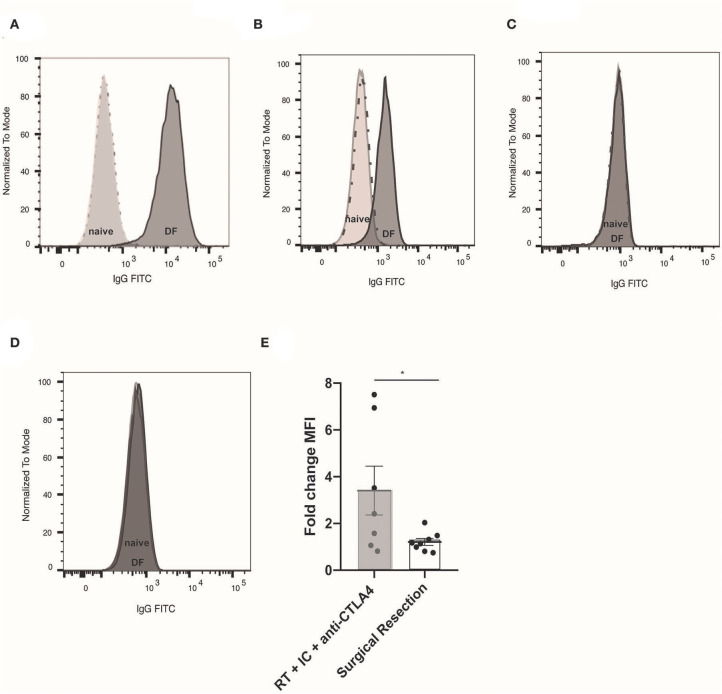
Tumor specific, adaptive humoral response observed after RT + IT-IC + anti-CTLA-4. **(A)** Flow cytometry histogram of B78 cells (unlabeled cells represented by dotted line) labeled with naïve serum (light gray) from a non-tumor bearing, untreated mouse, or serum from a mouse rendered disease free (DF) following RT + IC + anti-CTLA4 (dark gray). Serum was collected 10 days after 2 million B78 tumor cell were implanted by subcutaneous injection to the right flank of mice previously rendered DF of B78 tumors following RT + IC + anti-CTLA4. Unlabeled B78 cells and naïve serum labeled cells appear superimposed due to low naïve serum binding activity. **(B)** Flow cytometry histogram of cells only (dotted line), naïve serum (light gray), and RT + IC + anti-CTLA4 DF rendered serum (dark gray) binding to B16 (GD2-) melanoma; **(C)** Binding to PancO2 (GD2+) pancreatic cancer; and **(D)** Binding to PancO2 (GD2-), pancreatic cancer. In **(C,D)**, histogram of cells only (dotted line), and the histograms representing serum labeled cells appear superimposed due to serum binding activity being comparable to the background with no serum. **(E)** Fold change Median fluorescence intensity (MFI) of endogenous anti-tumor antibody from individual mice bound to B16 (GD2-) melanoma, representing change from pre-re-challenge to 10 days after re-challenge. Serum was obtained from mice rendered DF by RT + IC + anti-CTLA4 (gray box) or surgical resection (white box) prior to 2 million B78 melanoma tumor cell re-challenge (pre-re-challenge), or 10 days after re-challenge (post-re-challenge). Data in **(A–D)** represent one of three replicates; data in **(E)** is composite data from two replicate studies. *p < 0.05.

In order to assess for a humoral memory response, we collected serum from mice cured of B78 melanoma by an RT + IT-IC + anti-CTLA-4 *in situ* vaccine regimen and subsequently demonstrated immune memory by rejection of subcutaneous re-engraftment of B78 cells 90 days after treatment. Serum was also collected from naïve, non-tumor bearing C57BL/6 mice. Serum was incubated with B78 cells *in vitro* and the presence of anti-tumor antibodies was assessed using an anti-mouse IgG specific secondary antibody. Using flow cytometry to compare antibody labeling for B78 cells only, B78 cells incubated in serum from naïve mice (non-tumor bearing, untreated), and B78 cells incubated in serum from mice rendered disease-free with RT + IC + anti-CTLA-4, we observed the presence of endogenous anti-tumor antibodies against B78 in serum from disease-free mice (Figure 1A). To evaluate the specificity of these anti-tumor antibodies, we took the same serum and incubated with B16 GD2- melanoma cells (Figure 1B), Panc02 GD2+ pancreatic cells (Figure 1C), and Panc02 GD2- cells (Figure 1D). B16 is a parental melanoma line from which B78 was derived by expressing the GD2 target antigen for hu14.18-IL2. Serum antibodies from treated mice displayed recognition of B16 and B78 but did not show binding to Panc02 or Panc02 GD2+, demonstrating that mice rendered disease-free following *in situ* vaccination exhibit an adaptive, tumor-specific, humoral response. Detection of serum antibodies bound to B16 was reduced when decreasing amounts of serum sample were added to B16 cells, indicating a dilutional effect and further supporting specificity of the antibody-cell interaction (Supplemental Figure 3).

In order to determine whether this tumor-specific memory response was treatment-specific, mice bearing B78 flank melanomas were treated by surgical resection and monitored until D90 after surgery, at which point they were declared disease-free if no tumor recurrence was observed by clinical exam. Concurrently, we treated a separate cohort of mice with RT + IT-IC + anti-CTLA-4. All disease-free mice were re-challenged with subcutaneous re-engraftment of B78 cells at D90 after the start of treatment. Serum was collected prior to and 10 days after B78 re-challenge. Using flow cytometry, we tested for the presence of anti-tumor antibody in these serum specimens and observed the fold change in MFI of anti-tumor antibodies was significantly higher in RT + IT-IC + anti-CTLA-4 treated mice compared those cured by surgical resection (*n* = 7–8; *p* = 0.0401) (Figure 1E).

### Timing, Isotype, and Function of the Endogenous Antibody Response to RT + IT-IC + anti-CTLA-4

**Figure 2:**
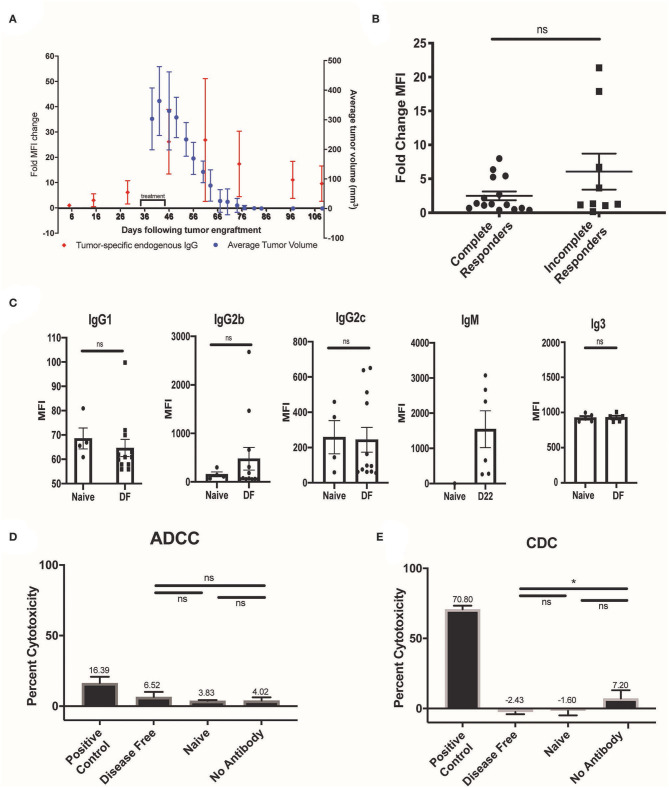
Timing, isotype, and function of the endogenous antibody response to RT + IT-IC + anti-CTLA-4. **(A)** Serum was collected from mice (*N* = 5) treated with RT + IC + anti-CTLA4 before, during, and after treatment and was tested for anti-tumor antibody binding to B16 melanoma (red dots). Corresponding mean tumor volume (for these same mice, *N* = 5) is represented by the blue dots with standard error of the mean (SEM). **(B)** Fold change MFI of serum collected from tumor bearing mice prior to treatment and at D22 after treatment in B78 tumor bearing mice. All mice were treated with RT + IC + anti-CTLA4; data shown represents samples from 4 separate experiments (*n* = 16 complete response, *n* =9 incomplete responses). Response to treatment was assessed at D90 after radiation, and serum was collected for analysis at this time. There was no significant difference in the fold change of MFI between the complete responders and the incomplete responders (*p* > 0.05). **(C)** DF, post re-challenge serum was analyzed by flow cytometry for the presence of IgG1, IgG2b, IgG2c, and IgG3 antibody subclasses that bound to B16, and is represented as MFI. D22 serum was analyzed by flow cytometry for the presence of IgM antibody that bound to B16, and is represented as MFI. D22 was chosen due to the propensity for IgM to rise early during the immune response. **(D)** ADCC capacity of disease free serum was tested using ^51^chromium-release assays with B16 GD2+ melanoma cells, murine effectors (effector to target ratio 50:1), and serum (*n* = 3 for naive, *n* = 15 for disease free), or ch14.18K322a (positive control) was added. Antitumor functionality was quantified by percent cytotoxicity and is represented by the mean and SEM for data obtained using 50 effector cells per target. **(E)** CDC was also measured, using anti-GD2 antibody 14G2a as a positive control. Data in **(D,E)** represent one of two replicates. *P* < 0.05 were considered significant and are indicated in figures *p < 0.05, NS, non-significant (*p* ≥ 0.05).

Serum was collected from mice bearing B78 melanoma tumors before, during, and after RT + IT-IC + anti-CTLA-4 treatment in order to determine the time course of the anti-tumor adaptive humoral response. Flow cytometry was performed to test for binding of endogenous serum antibodies to B16 melanoma cells, which were used to eliminate potential confounding from the presence of the therapeutic hu14.18-IL2 IC in serum during treatment. These studies demonstrated that a small amount of tumor-specific endogenous antibody was present after tumor engraftment and prior to treatment and this markedly increased with the initiation of *in situ* vaccine therapy (Figure 2A). No significant correlation was observed between the pre- to post-treatment fold-change in B16 cell labeling by serum tumor-specific antibodies and complete response to *in situ* vaccination (*p* = 0.4460), although animals with the highest increase in humoral response following *in situ* vaccination were among the non-complete responders (Figure 2B). Response to treatment was assessed at D90 after radiation; a complete response was recorded if no tumor was detectable, and serum was collected for analysis at this time. Data shown represent samples from four separate experiments (*n* = 16 complete response, *n* = 9 incomplete responses).

Using flow cytometry, we evaluated the IgG isotypes composing the humoral response to *in situ* vaccination. For this, we labeled B16 cells using serum from mice rendered disease-free from a B78 melanoma tumor by treatment with RT + IT-IC + anti-CTLA-4 and demonstrating immune memory by rejection at D90 of re-implantation with B78. These B16 cells were labeled further with isotype-specific secondary antibodies to confirm the presence of IgM, IgG1, IgG2b, IgG2c, and IgG3 antibody subclasses (Figure 2C). IgG2a, while a very important antibody isotype for ADCC, is not generated in C57bL6 mice (23) and thus we did not include this antibody in our panel. Given the potential effector functions of murine IgG2b antibodies in the mediation of ADCC and CDC (20, 24, 25), we pursued *in vitro* assessment of the functionality of the endogenous serum antibodies we observed in mice rendered disease-free by RT + IT-IC + anti-CTLA-4. ADCC was tested using ^51^chromium-release assays as previously described (20). No significant increase in cytotoxicity was observed in the disease-free serum samples compared with the naïve serum treated samples (*p* > 0.05) (Figure 2D). CDC was similarly measured by co-incubation of labeled target cells with rabbit complement C1 and serum. No significant increase in CDC was observed in the presence of disease-free serum, as compared with the naïve serum (*p* > 0.05) (Figure 2E).

### B Cells Are Not Necessary for the *in situ* Vaccine Effect of RT + IT-IC + anti-CTLA-4

**Figure 3:**
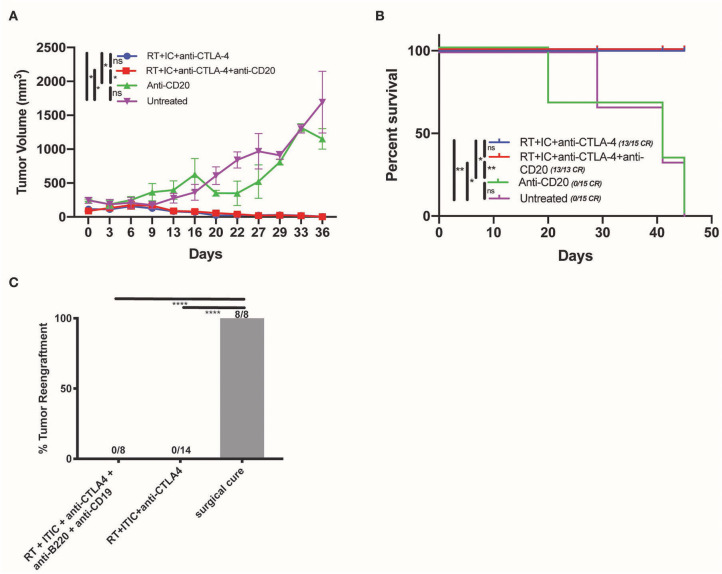
B cells are not necessary for the anti-tumor effect of RT + IT-IC + anti-CTLA-4. **(A)** Average tumor volume growth curves (starting size 200 mm^3^) of mice treated with RT + IC + anti-CTLA-4, RT + IC + anti-CTLA-4 + anti-CD20, anti-CD20, or untreated controls. Mean and SEM are shown. **(B)** Kaplan Meier survival curves of mice treated in **(A)**. **(C)** Tumor engraftment of B78 administered at D90 to test for immune memory for DF mice cured by surgical resection (8/8 regrew tumors), RT + IC + anti-CTLA4 (0/14 regrew tumors), and RT + IC + anti-CTLA4+anti-CD19+anti-B220 (0/8 regrew tumors). Data shown represents one of three replicates. *P* < 0.05 were considered significant and are indicated in figures as ****p < 0.00001, **p < 0.001, *p < 0.05, NS, non-significant (*p* ≥ 0.05).

To determine the necessity of B cells in the therapeutic effect of RT + IT-IC + anti-CTLA-4 in mice bearing a B78 melanoma tumor, mice were depleted of B cells. This was performed by IP injection of anti-CD20 on D0 and D13 of treatment, in order to deplete naïve B cells which might be educated to recognize tumor antigens during treatment. Depletion was confirmed by flow cytometry on blood samples obtained on D1 as described in the methods (Supplemental Figure 1). As a secondary confirmation of the effective depletion of anti-tumor antibody producing B cells, we quantified the production of anti-tumor antibodies from B cell-depleted and non-depleted groups. We observed that endogenous anti-tumor antibody levels were reduced in mice that received B cell depletion relative to mice that had no B cell depletion (*p* < 0.05) (Supplemental Figure 4). B cell depletion did not affect either the growth rate of untreated B78 melanoma or the response of these tumors to RT + IT-IC + anti-CTLA-4 (Figure 3A). Mean tumor size did not differ significantly between RT + IT-IC + anti-CTLA-4 and RT + IT-IC + anti-CTLA-4+anti-CD20 groups (*p* = 0.7002) or untreated and anti-CD20 treated groups (*p* = 0.9188). Over three replicate studies, RT + IC + anti-CTLA-4 treatment group resulted in 13/15 complete responses, RT + IC + anti-CTLA-4 + anti-CD20 resulted in 13/13 complete responses. No complete responses were observed in the untreated or anti-CD20 treatment groups (Figure 3A). Overall survival also was not significantly different between RT + IT-IC + anti-CTLA-4 and RT + IT-IC + anti-CTLA-4 + anti-CD20 groups (*p* > 0.9999), or between the untreated and anti-CD20 treated groups (*p* = 0.9122) (Figure 3B).

We evaluated the impact of B cell depletion on RT + IT-IC + anti-CTLA-4—induced generation of immunologic memory in mice rendered disease-free by this treatment. To achieve this, we depleted such mice of both plasma cells and B cells by IP injection of using anti-CD19 and anti-B220 beginning on D69 after initiation of treatment (21 days before the re-challenge on D90). Anti-CD19 and anti-B220 were selected in accordance with the literature (21) in order to ensure depletion of B cell subsets which do not express CD20. We confirmed cell depletion by flow cytometry as described in the methods. Twenty-one days after depletion and 90 days after treatment initiation, these mice were tested for immunologic memory by re-implantation with 2 million B78 melanoma cells in the subcutaneous tissue on the flank, contralateral to the initial tumor site, and monitored for tumor rejection. Consistent with the presence of robust immunologic memory that was not dependent on B cells or tumor-specific antibody, no tumor engraftment was observed in mice rendered disease-free with RT + IC + anti-CTLA-4 (0/14) or in those rendered disease-free with RT + IC + anti-CTLA-4 and subsequently depleted of B cell and plasma cells by anti-CD19 + anti-B220 (0/8) (Figure 3C). All control mice rendered disease free from a B78 tumor by surgical resection, showed tumor engraftment and growth when re-implanted with the same number of B78 cells 90 days after surgical resection (Figure 3C), despite the presence of antitumor antibody in these mice made disease free by surgical resection (Figure 1E).

### Co-incubation of Disease Free Serum With B16 Cells Does Not Delay B16 Lung Metastases Engraftment

**Figure 4:**
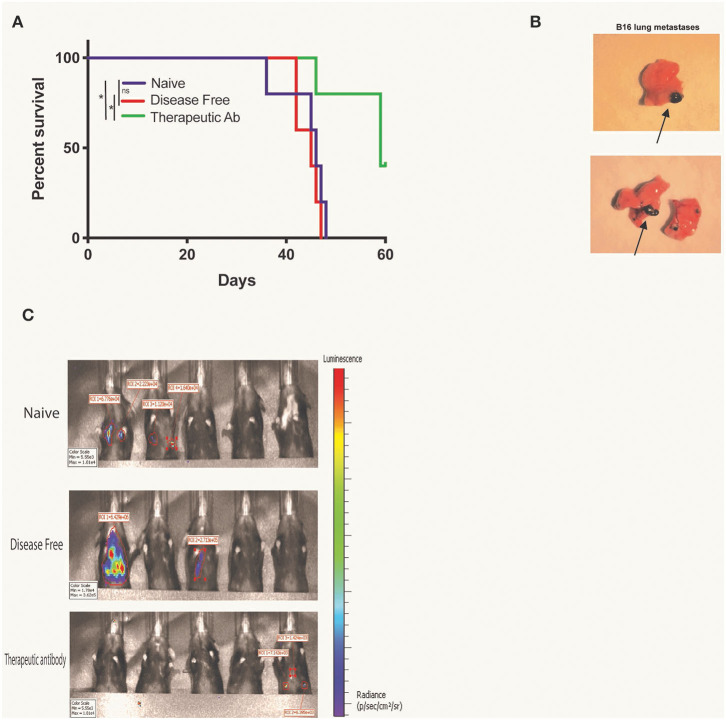
Presence of disease free serum does not delay B16 lung metastases engraftment. **(A)** Kaplan Meier survival curve for mice with B16-GD2+luc+ lung metastases. Fifty thousand B16-GD2+luc+ cells were incubated with naïve serum, DF serum, or hu14.18K322A therapeutic antibody prior to intravenous injection in mice. Mice were sacrificed when clinical signs of dyspnea were observed and necropsy was performed for confirmation of **(B)** lung metastases (lungs representative of naïve, left, and DF, right, serum groups, metastases distinguished by black arrows). **(C)** On day 28 after tumor engraftment, all mice were imaged by a Perkin Elmer IVIS Spectrum for the presence of B16luc+ lung metastases (black arrows). Data represents one of three replicates. *P* < 0.05 were considered significant and are indicated in figures as *p < 0.05, NS, non-significant (*p* ≥ 0.05).

Finally, we tested for potential therapeutic anti-tumor effects of endogenous anti-tumor antibodies in the serum of mice rendered disease-free by RT + IT-IC + anti-CTLA-4 treatment of a B78 tumor. For this purpose, B16 GD2+ Luc+ cells were incubated in serum that had been collected from naïve mice or during the memory phase of mice rendered disease-free by RT+IC+anti-CTLA-4. As a positive control, we used serum from naïve mice in which we directly added the therapeutic anti-GD2 antibody, hu14.18K322A. Cohorts of syngeneic C57BL/6 mice were then IV injected with 50,000 cells/mouse from these respective serum-labeled B16 GD2+ Luc+ cells, mimicking a setting of circulating tumor cell metastases. Labeling of these B16 cells with serum from disease-free mice did not increase survival of mice (Figure 4A), compared with naïve serum labeling of B16 cells. On the other hand, serum with added therapeutic anti-GD2 hu14.18K322A antibody did prolong mouse survival when it was used to pre-label these same B16 cells prior to IV injection (*p* < 0.05). Mice from each of these cohorts were sacrificed when clinical signs of dyspnea were observed and necropsy was performed to confirm the presence of metastases with predominant burden observed in lung (Figure 4B). Additionally, all mice were imaged by a Perkin Elmer IVIS Spectrum on D28 after tumor engraftment to assess bioluminescence from B16 GD2+ Luc+ metastases. Representative images are shown from each treatment group upon necropsy (Figure 4C).

## Discussion

We demonstrate an endogenous tumor-specific antibody response following a therapeutic regimen that combines RT + IT-IC + anti-CTLA-4 for the treatment of a syngeneic B78 melanoma. While our treatment regimen includes an exogenous antibody-based therapeutic (hu14.18-IL2 IC) targeting the GD2 antigen on these melanoma cells, the endogenous humoral response generated following this treatment is not specific to GD2 and recognizes antigens shared by B78 and the B16 melanoma line that is parental to B78 but lacks GD2. We also observe a tumor-specific endogenous antibody response in tumor-bearing mice that have not received the vaccine; however, the magnitude of the response is increased following the treatment regimen (Figure 2A).

Interestingly, mice that are rendered disease-free by either *in situ* vaccine or by surgical resection exhibit an endogenous anti-tumor antibody response; however, the response to tumor re-engraftment differs between these groups. Surgically cured mice do not display immunologic memory as they do not reject tumor re-challenge (Figure 3C) and show little change in the level of tumor specific antibody production in response to tumor cell re-challenge (Figure 1E). In contrast, *in situ* vaccine treated mice show robust immunologic memory capable of potent rejection of tumor re-challenge (Figure 3C) and a demonstrable increase in the production of tumor-specific antibody following tumor cell re-implantation (Figure 1E). We propose a T-cell dependent, B cell activation driving the production of IgG anti-tumor antibodies during *in situ* vaccine, resulting in the establishment of a humoral memory response not seen in mice cured of tumor by surgical resection alone, despite the presence of identical tumor antigens during the treatment phase. Interaction of naïve B cells with T helper cells through linked recognition of a shared antigen coordinate a cytokine response resulting in clonal proliferation of B cells, and later, differentiation into memory B cells (26–28). Memory anti-tumor antibodies produced by *in situ* vaccine treated mice, and not by surgically cured mice, suggest differences in the composition of the immune environment of the tumor following these two distinct treatments. Radiation may increase release of antigen within the tumor, including those perhaps better suited for B cell recognition (29). Anti-CTLA4 reduces regulatory T cells, allowing for increased T cell activation. The presence of IL2 within the tumor through intratumoral IC may have a crucial role in driving this proposed T-dependent antibody response, as IL2 has been shown to be critical for CD40-mediated activation of naïve B cells by CD4 T cells (30). Though both surgery and *in situ* vaccine treatments result in the elimination of the primary tumor, confirmation of a B cell memory response following *in situ* vaccination supports the hypothesis that a powerful, T cell driven immune activation persists long after treatment has ended. Here we report that the T cell-driven, anti-cancer memory response to our *in situ* vaccine regimen is not unidimensional, and results in B cell activation leading to establishment of tumor-specific humoral memory.

While the level of tumor-specific antibody produced following treatment did not correlate with complete tumor response (Figure 2B), the antibody observed in the post-disease eradication setting may serve as an early biomarker of adaptive recognition of tumor following immunotherapy. We observe an increase of anti-tumor IgG after tumor rechallenge in our mouse model, even in the absence of palpable engraftment, suggesting that these memory derived anti-tumor antibodies may fluctuate with the presence of increasing tumor burden, even if it is not yet detectable macroscopically. The potential use of serum anti-tumor antibodies as a means of monitoring microscopic tumor burden warrants further exploration. We have now opened a prospective clinical study testing the safety and efficacy of the RT + IT-IC + anti-CTLA-4 *in situ* vaccine regimen in patients with metastatic melanoma (NCT03958383) and the potential role of the endogenous anti-tumor humoral response as a biomarker will be further investigated as part of that study.

Investigation of endogenous anti-tumor antibodies may also provide enable novel approaches to the identification of tumor-specific antigens targeted by the humoral response. This could provide new targets for potential therapeutic agents and advance our understanding of humoral anti-tumor responses. It is largely unknown how the repertoire of tumor antigens recognized by an endogenous humoral response may compare to that recognized by an adaptive T cell response. This model system will now facilitate direct comparative studies in this regard and we are now pursuing such objectives by using peptide array binding assays to identify tumor antigens recognized by the endogenous humoral response. Comparing these antibody-recognized tumor antigens with those identified by investigation of the MHC peptidome and T cell receptor antigen specificity will begin to shed light on the potential interaction of adaptive B and T cell responses against tumor. Furthermore, where T cells do not effectively recognize tumor antigens identified as targets of humoral immunity, such tumor-specific antibodies might be utilized in the custom design of personalized cell therapies such as chimeric antigen receptor (CAR) T cells.

In our studies, B cell depletion does not influence the growth of B78 melanoma tumors, the tumor response to RT + IT-IC + anti-CTLA-4, or the immunologic memory established by this treatment (Figure 3). Previous studies demonstrated that the therapeutic efficacy and immunologic memory established by RT + IT-IC + anti-CTLA-4 is dependent on T cells (9). Our data indicate that B cells are not necessary for effective treatment response to the RT + IT-IC + anti-CTLA-4 *in situ* vaccine regimen. We do not observe a rise in anti-tumor antibody detectable in the serum against B78 tumors after tumor re-challenge in mice cured of B78 by RT + IT-IC + anti-CTLA-4 after B cell depletion (Supplemental Figure 4), confirming a lack of antibody-based humoral memory response in B cell deficient mice. In this specific case, these antibodies are not necessary for tumor re-challenge rejection. However, it is very possible these antibodies do have a role that may add to the overall anti-tumor response, but absence of this endogenous antibody response does not translate to clinical failure of rejection in the face of a robust T cell response. These data do not exclude a potential role for B cells in this adaptive immune including the possibility that this regimen could be further augmented using therapies that target B cells, and enhance their role in antigen presentation (31).

We were unable to identify a functional or therapeutic capacity of the endogenous tumor-specific antibody response generated by treatment of B78 tumor bearing mice with RT + IT-IC + anti-CTLA-4 (Figures 2, 4). In previous studies, we have shown B78 cells are susceptible to ADCC and CDC (32), thus we do not suspect B78 tumor resistance mechanisms to be responsible for the lack of observed protective effect in this model system. In these experiments, the maximum amount of serum we could allocate from our stock and still have enough for a proper number of replicates was used. However, it is important to recognize the limitations in quantifying serum levels of a polyclonal endogenous anti-tumor antibody mixture. One shortcoming of investigations of this nature is the inability to report an antibody titer by means of ELISA, as the antibodies in this study are comprised of a polyclonal population with multiple antigenic targets. By selecting one antigen for an ELISA assay, we would inadvertently exclude a subgroup of the population of antibodies generated against B78. Thus, we are unable to report a traditional titer, which would otherwise provide insight into the strength of protective response to expect for a particular antibody response. Without this information, it is important not to exclude the possibility that an anti-tumor effect of this antibody could be identified, perhaps under different conditions using higher concentrations of antibody or volume of serum antibody or in different incubation environments. Using the maximum sample available to us, provided constraints in quantities of serum, we were unable to detect an ADCC response in DF serum (with anti-tumor Abs present) relative to naive serum (which lacks anti-tumor Abs). It is very possible the level of exogenous antibody used as a positive control (ch14.18) is much higher than the level of endogenous anti-tumor antibodies in the serum, such that the exogenous antibodies are able to overcome any moderate mechanisms of tumor resistance, while the less concentrated endogenous antibodies are not.

Due to the presence of antibody subclasses (IgG2b and IgG2c) associated with Th1 anti-tumor responses, we suspect that the endogenous tumor-specific humoral response in these mice may be generated in the process of developing a therapeutically meaningful adaptive T cell response, even if the anti-tumor antibody is not directly involved in that *in vivo* anti-tumor destruction. This may explain why some of the highest levels of tumor-specific antibody detected following this treatment regimen occurred in mice that did not experience a complete tumor response (Figure 2B). It is tempting to speculate that these mice may have developed more of a Th2 anti-tumor immune response, resulting in reduced anti-tumor efficacy and greater production of anti-tumor antibody. If confirmed, future studies will investigate what factors may lead to such modulation of response in these syngeneic tumor models. Understanding the antigenic targets of a Th2 skewed immune response, and comparing these targets with those recognized during a Th1 skewed response, may further provide insight into the immune targets required to generate a protective T cell driven anti-tumor response. These important studies are currently underway.

Here, we present evidence of a robust, long lasting, tumor-specific humoral immune response provoked by RT + IT-IC + anti-CTLA-4 *in situ* vaccination in a syngeneic murine melanoma model. In characterizing this response, we provide a model system that others and we can use to investigate this unique, treatment-dependent adaptive humoral response to an otherwise T cell-mediated therapy. Such studies will be helpful in more broadly understanding and effectively engaging the systemic anti-cancer adaptive immune response.

## Data Availability Statement

The datasets generated for this study are available on request to the corresponding author.

## Ethics Statement

All animal studies were performed after approval by the University of Wisconsin-Madison Institutional Animal Care and Use Committee (IACUC).

## Author Contributions

CB was responsible for primary design and execution of experiments with experimental design input from RP, AR, PS, and ZM. ZM was the primary investigator and ultimately determined the direction of experimental questions. CH, ES, RS, CS, and AP assisted with mouse work for all studies. AP and SG assisted with B cell depletion experiment design and quality checks. JH conducted cytotoxicity assays (ADCC and CDC) with the help of CB. AH and PC provided assistance in designing and conducting flow cytometry experiment panels and troubleshooting. SG synthesized and provided anti-CD20 antibody for experiments, provided input on experimental design, and execution for mouse work. AE was responsible for quality checks of tumor cells lines and inducing expression of GD2+ and luc+ for experimental use. All authors contributed to the article and approved the submitted version.

## Conflict of Interest

SG was employed by the company Provenance Biopharmaceuticals. The remaining authors declare that the research was conducted in the absence of any commercial or financial relationships that could be construed as a potential conflict of interest.
