# Supplementary figures and images for: In situ Vaccine Plus Checkpoint Blockade Induces Memory Humoral Response
Source: Front Immunol. 2020 Jul 24;11:1610. doi: 10.3389/fimmu.2020.01610 (PMC7396490; doi:10.3389/fimmu.2020.01610)

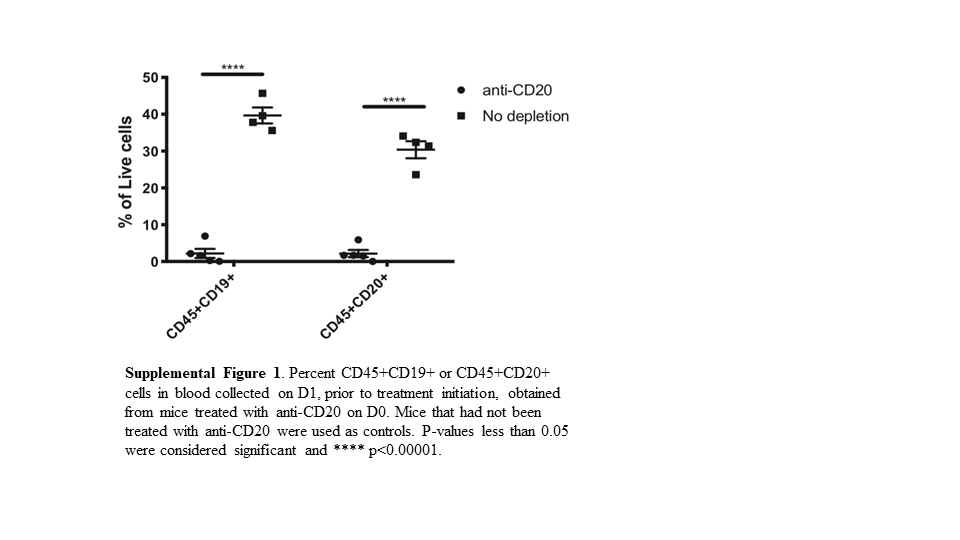

Supplement: Supplementary file 1 [file Image_1.TIF]

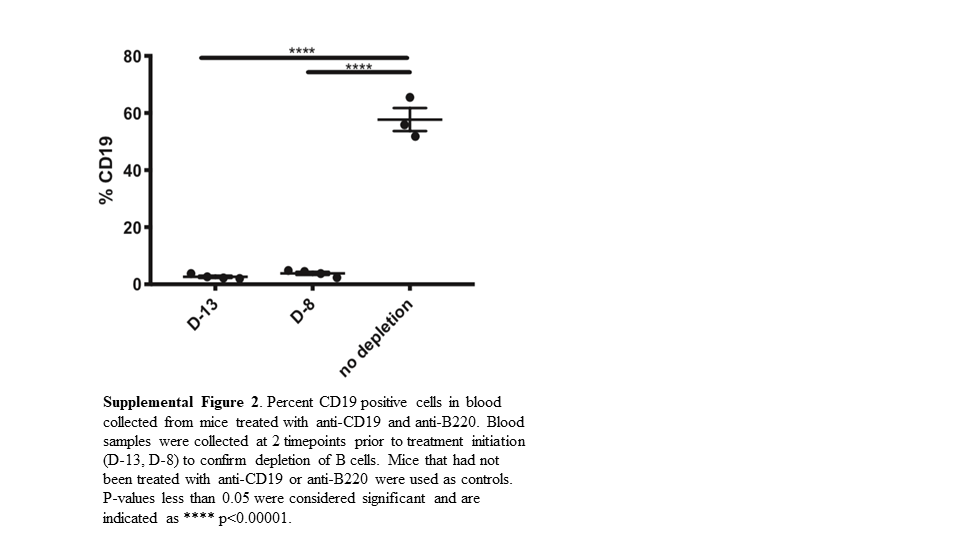

Supplement: Supplementary file 2 [file Image_2.TIF]

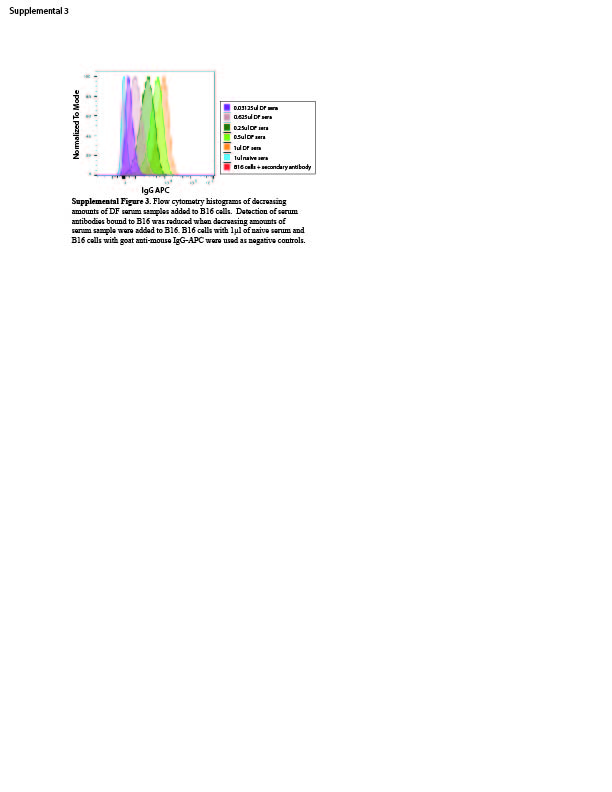

Supplement: Supplementary file 3 [file Image_3.jpg]

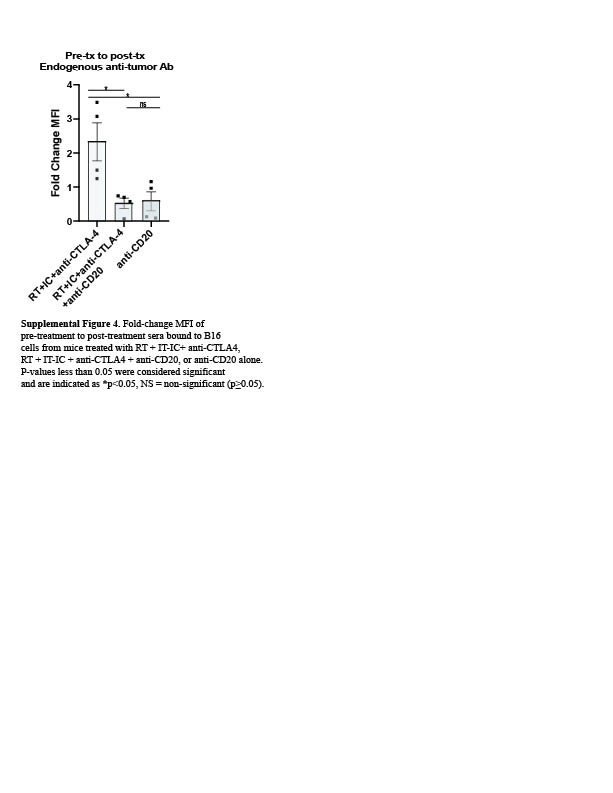

Supplement: Supplementary file 4 [file Image_4.JPEG]
